# Deploying Indocyanine Green Fluorescence-Guided Navigation System in Precise Laparoscopic Resection of Pediatric Hepatoblastoma

**DOI:** 10.3390/cancers14246057

**Published:** 2022-12-09

**Authors:** Ronglin Qiu, Yaohao Wu, Jianhang Su, Luping Chen, Minyi Liao, Zhuangjie Zhao, Zijie Lu, Xiangang Xiong, Shikai Jin, Xiaogeng Deng

**Affiliations:** Department of Pediatric Surgery, Sun Yat-Sen Memorial Hospital, Sun Yat-Sen University, Guangzhou 510120, China

**Keywords:** hepatoblastoma, indocyanine green, fluorescence-guided system, laparoscopic surgery

## Abstract

**Simple Summary:**

Indocyanine green (ICG) is a safe reagent that has been clinically approved to assist in intraoperative navigations for the resection of hepatocellular carcinoma. Although an increasing trend has been observed in the use of an ICG fluorescence-guided system for hepatoblastoma (HB) surgery, studies involving a larger cohort of children is still lacking, which indicates a limitation in the application of ICG for pediatric HB surgery. Furthermore, there have also been few reports on its application in the field of laparoscopic surgery. Therefore, seven cases of HB resection were performed using the fluorescence-guided laparoscopic system in this study, and their clinical data were analyzed retrospectively to clarify the characteristics and outcomes of intraoperative ICG fluorescent imaging in laparoscopic HB resection among children.

**Abstract:**

*Background*: Hepatoblastoma (HB) is the most common form of liver cancer in children. To date, complete tumor resection is still the gold standard for treating HB. Indocyanine green (ICG) has been identified as a sensitive adjunct that is highly effective in the identification and surgical management of local and metastatic HB. It has thus becomes an increasingly popular choice among surgeons in HB resection surgeries that are fluorescence-guided. However, laparotomy remains the preferred choice in most cases since the applications and limitations of fluorescence-guided laparoscopic surgery in treating HB remain unclear. In this study, the characteristics and outcomes of laparoscopic HB resections that were guided by intraoperative ICG fluorescent imaging were investigated. *Methods*: Seven HB patients underwent ICG-guided laparoscopic HB resection surgery from August 2019 to December 2021. ICG was intravenously administered to the patients at a dosage of 0.5 mg/kg 48 h prior to the scheduled operation. During operation, tumor localization and resection boundary were guided by fluorescence visualization. The data on surgical and clinical features were collected retrospectively. *Results*: The resection area and tumor boundary could be clearly viewed in real-time under the ICG fluorescence imaging navigation system during operation, except for one patient who had received interventional chemoembolization before surgery. The image produced by laparoscopic fluorescence navigation was clear since it was not affected by ambient light. All tumors were completely resected as confirmed by negative margins for HB during postoperative pathological examination. No residual or recurrence were also found through computed tomography during follow-up visits from 9 to 37 months. *Conclusions*: ICG fluorescence-guided laparoscopic surgery is safe and effective in treating HB due to its ability to provide clear information on tumor localization and delineate tumor margins in real-time.

## 1. Introduction

Hepatoblastoma (HB) is the most common form of malignant hepatic tumor in children [1]. With advances in imaging techniques, neoadjuvant chemotherapy, surgery, and postoperative chemotherapy, the overall survival rate for children with HB has greatly improved. Complete surgical resection of tumors remains the most critical intervention required to achieve a long-term cure for HB patients. Although preoperative radiographic imaging, ultrasound, visual inspection, and palpation can identify lesions, margins, and vascular or local invasion, real-time guided methods with high specificity are still lacking [2,3]. Real-time methods that are highly sensitive in identifying tumor boundaries, residual lesions, and grossly unidentifiable tumors will not only enhance the accuracy of tumor resection, but also improve tumor outcomes, enabling surgeons to perform R0 resection.

Indocyanine green (ICG) is a dye that accumulates in liver tumor tissues to show clear fluorescence images with strong contrast between cancerous and normal liver tissues [4,5]. It is currently being used in adult hepatobiliary surgery for the visualization of target lesions or liver segments, allowing surgeons to conveniently identify liver tumors or perform precise anatomical resection during open and laparoscopic hepatectomy [6]. Since HB possesses similar features to hepatocellular carcinoma, the ICG fluorescence-guided system has been increasingly applied in HB surgery [7]. Subsequent reports of ICG fluorescence-guided surgery for HB mainly include local tumor, liver, or lung metastasis lesions, residual, and recurrence tumors [8,9,10,11]. Yamada et al. and Cho Y.J. et al. applied ICG fluorescence imaging as a method of surgical navigation in detecting HB lesions and successfully demonstrated its safety and usefulness [9,11]. Meanwhile, the high sensitivity of ICG imaging in guiding pulmonary metastasectomy was revealed by Misa et al. through their study [12]. Another study also reported the effectiveness of ICG fluorescence imaging in HB resection and the detecting of small lesions not shown in preoperative imaging [13]. Laparoscopy is increasingly favored by surgeons in pediatric tumor surgery for its benefits of minimal invasiveness, fast recovery, and aesthetic advantage, features that make early postoperative chemotherapy possible. However, few reports are currently available for the use of ICG fluorescence-guided laparoscopic surgery in treating HB. Henceforth, the characteristics and outcomes of intraoperative ICG fluorescence-guided laparoscopic HB resection were explored in this study.

## 2. Materials and Methods

Seven children (six males and one female, aged 13 days–36 months) with HB who were admitted to the Department of Pediatric Surgery at Sun Yat-Sen Memorial Hospital, Sun Yat-Sen University from August 2019 to December 2021 were retrospectively reviewed. They received preoperative chest and abdominal CT scan to evaluate the following details: tumor position, size, relationship with surrounding blood vessels, and the occurrence of distant metastasis. Alpha-fetoprotein (AFP) levels of all patients were checked before and after surgery. The condition of all patients involved in this study were confirmed by B-mode ultrasound-guided biopsy. Before surgery, the chemotherapy regimen was formulated according to the “2016 Chinese Children Cancer Group Hepatoblastoma Multidisciplinary Diagnosis and Treatment Expert Consensus”. Five patients received neoadjuvant chemotherapy (four patients received four cycles of C5VD (chemotherapy of cisplatin, 5-fluorouracil, vincristine, and doxorubicin), whereas the other patient received three cycles of cisplatin combined with doxorubicin, and two cycles of ifosfamide, carboplatin, and etoposide), one patient received three cycles of C5VD chemoembolization in another hospital before admission, and one case did not receive neoadjuvant chemotherapy. All seven patients underwent laparoscopic HB resection using the ICG fluorescence-guided navigation system.

ICG (Yichuang, Dandong, China) was administered intravenously to the patients at a dosage of 0.5 mg/kg 48 h prior to the operation. Informed consent from parents of the patients involved were obtained before operation. The fluorescence endoscopic navigation system (Optomedic, Foshan, China) was adopted for ICG fluorescence imaging during operation. This system provides four imaging modes: high-definition white light, standard fluorescence, color fluorescence, and multi-mode fluorescence. It enables the visualization of invisible near-infrared rays through the monitor and enhances visualization of tissue perfusion at real-time during operation.

A trocar was inserted using the four-hole method. After entering the abdominal cavity, the Optomedic fluorescence imaging system was turned on to check the liver in high-definition white light mode, before being switched to the standard fluorescence mode to check the location of the tumor with green fluorescence imaging. The round ligament, falciform ligament, and coronary ligament were broken with a Harmonic scalpel. The first hepatic hilum was exposed, and the corresponding hepatic vessels, portal veins, bile ducts, or branches were freed and ligated according to the liver lobe or the segment intended to be resected. The electrocautery procedure was deployed to mark the cutting line on the surface of the liver about 2 cm away from the green fluorescent border. Adhesions around the tumor and the surrounding ligaments were broken with a Harmonic scalpel. The liver was resected along the cutting line using a Harmonic scalpel, whereas blood vessels and bile ducts of the liver parenchyma were ligated one by one with Hem-O-Locks. During operation, the cutting line was adjusted according to real-time fluorescence imaging. After complete resection of the tumor, the cut surface of the liver was electrocoagulated by means of electrocautery to stop the bleeding. Bile leakage and green fluorescence imaging on the liver section were subsequently checked. Among the patients involved in this study, four cases received partial hepatectomy, one case received right hemihepatectomy, one case received central bisegmentectomy, and one case received left lateral segmentectomy.

After surgery, five patients received four cycles of C5VD, one patient received two cycles of ifosfamide, carboplatin, and etoposide, and one patient received four cycles of C5V chemotherapy. Clinical data were collected including the time of surgery, intraoperative blood loss, and postoperative complications. All children received neoadjuvant chemotherapy, with the course of chemotherapy being decided based on the type of pathology and clinical stage. All patients had their follow-up visits to the outpatient clinic over a period of 9 to 37 months (average, 24 months) after their respective chemotherapy course, where their liver function parameters, AFP levels, abdominal CT, and whole-body PET-CT scans were recorded.

## 3. Results

Clinical data of the patients who underwent ICG fluorescence-guided laparoscopic surgery are shown in Table 1. There were six males and one female with an average age of 14 months (range, 13 days–36 months). Upon the first visit, five cases were diagnosed as stage III according to the PRETEXT system for pediatric liver tumor staging, while the other two cases were diagnosed as stage II and I, respectively. None of their tumors were metastatic. After four to five cycles of neoadjuvant chemotherapy, all cases were estimated as either POSTTEXT II or I. ICG fluorescence-guided laparoscopic surgery was successfully carried out in all cases. Four cases underwent segmental hepatectomy, one case underwent right hemihepatectomy, one case underwent mesohepatectomy, and the last case underwent left lateral hepatic lobectomy. The median size of the tumor was 65 mm (range, 25–87 mm).

**Figure 1:**
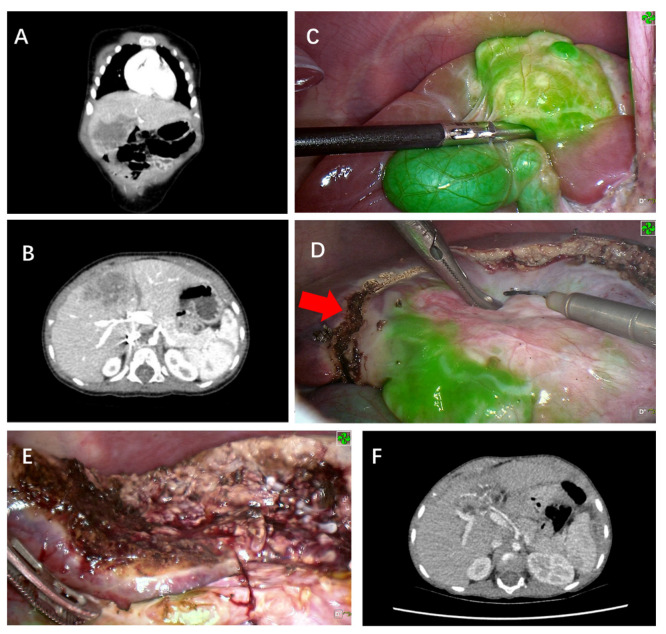
ICG fluorescence-guided laparoscopic hepatectomy of Segment 4a (case no. 5 in Table 1). (**A**) Preoperative coronary CT examination of tumor. (**B**) Preoperative cross-sectional CT examination to measure the size of the mass. (**C**) Green fluorescence was observed in the S4 segment of the liver and gallbladder after the fluorescence mode was turned on. (**D**) Before liver removal, a marker line (red arrow) was made on the surface of the liver based on fluorescence imaging (**E**). After complete removal of the tumor, the incision margin was checked and no fluorescence was observed, indicating the complete removal of the tumor without bile leakage. (**F**) No signs of tumor residues or recurrence were observed via CT 12 months after surgery.

**Figure 2:**
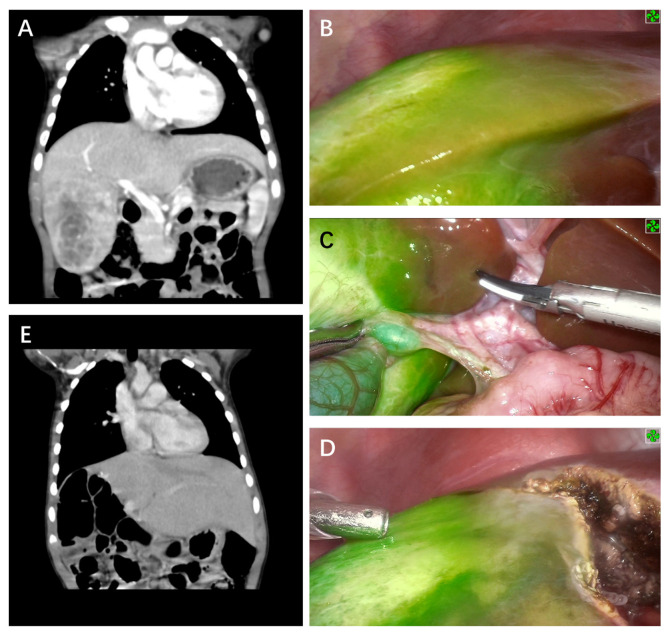
ICG fluorescence-guided laparoscopic right hepatectomy. (**A**) Preoperative CT image of coronal position and cross-section. (**B**) Tumor fluorescence was obvious during surgery with clear demarcation of normal tissue. (**C**) The relationship between tumor and biliary tract, hepatic artery, and portal vein were identified in fluorescence mode. (**D**) The marking line was continuously corrected according to fluorescence imaging during tumor resection. (**E**) No signs of tumor residues or recurrence were observed via CT 12 months after surgery.

**Figure 3:**
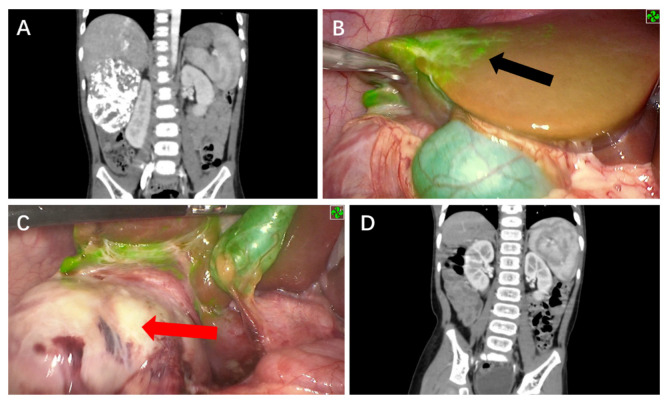
ICG fluorescence-guided laparoscopic hepatectomy of Segments 5, 6, and 7. (**A**) Preoperative CT image to assess tumor size and extent. (**B**) Irregular fluorescence was observed between the tumor and normal liver tissues in intraoperative fluorescence mode (black arrow). (**C**) No obvious fluorescence was seen in the tumor (red arrow). (**D**) No signs of tumor residues or recurrence were observed via CT 12 months after surgery.

**Figure 4:**
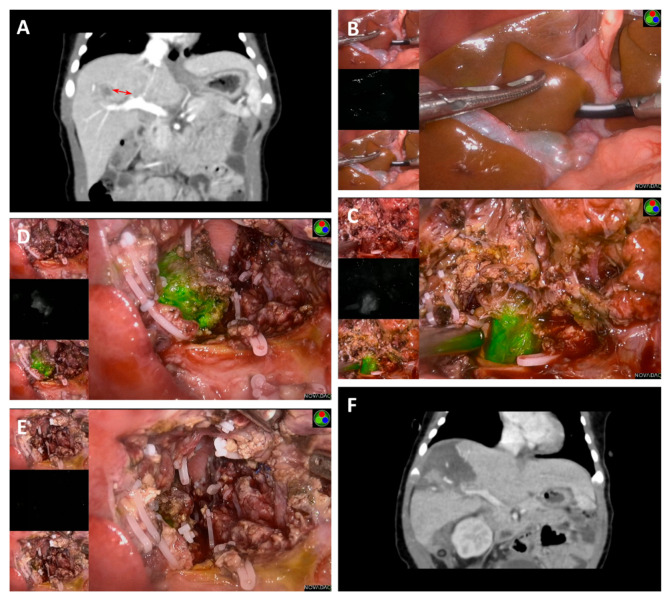
ICG fluorescence-guided laparoscopic hepatectomy of Segment 8. (**A**) Preoperative CT image shows that the tumor body was far away from the liver surface, about 1.24 cm from the hepatic hilar (red double arrow). (**B**) No fluorescence was observed on the surface of the liver in fluorescence mode. (**C**) The liver parenchyma around the hepatic hilar was chosen as the incision point based on preoperative CT images. Fluorescence was observed at a depth of approximately 0.5 cm after incision. (**D**) Tumors were isolated and removed according to real-time intraoperative fluorescence. (**E**) No fluorescence was seen on the liver surface after complete resection of the tumor. (**F**) No signs of the tumor residues or recurrence were observed via postoperative CT.

**Figure 5:**
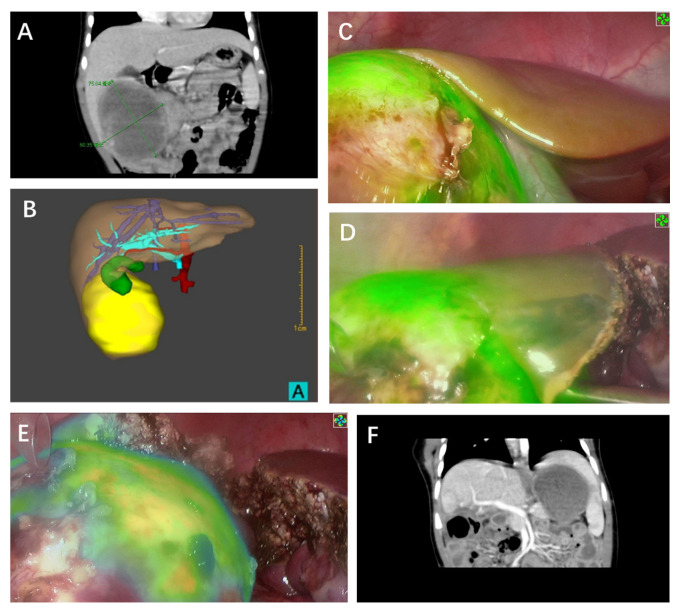
ICG fluorescence-guided laparoscopic hepatectomy of Segments 5 and 6. (**A**) Preoperative CT image to assess the tumor location and size. (**B**) Preoperative 3D imaging system to assess the relationship between the tumor, surrounding blood vessels, and bile ducts. (**C**). Tumor fluorescence was obvious during surgery with clear demarcation of normal tissue. (**D**) Marker line was made on the liver surface according to tumor fluorescence. (**E**) No fluorescence was observed on the incisal margin after resection. (**F**) No signs of tumor residues or recurrence were observed via postoperative CT.

Tumors found on the liver surface showed bright green fluorescence in real-time with a clear margin in contrast to normal liver tissue for five cases (Figure 1 and Figure 2). Interestingly, the case that was treated with chemoembolization before surgery did not show signs of tumor fluorescence (Figure 3). ICG imaging of the tumor in this case yielded a false-negative result (case no. 2). Another special case was case no. 6, whereby no green fluorescence was observed in the liver surface at first. The image of a green fluorescent tumor appeared only after 5 mm of incision into the liver parenchyma (Figure 4). This case demonstrated the limitation of ICG fluorescence in penetrating deep tissues. In addition, we performed preoperative three-dimensional imaging in order to assess the relationship between the tumor, surrounding blood vessels, and bile ducts (Figure 5). No tumor residual and bile leakage were detected in real-time during when ICG fluorescence-guided laparoscopy hepatectomy was completed. Postoperative pathological examination proved negative margins for all tumors.

**Figure 6:**
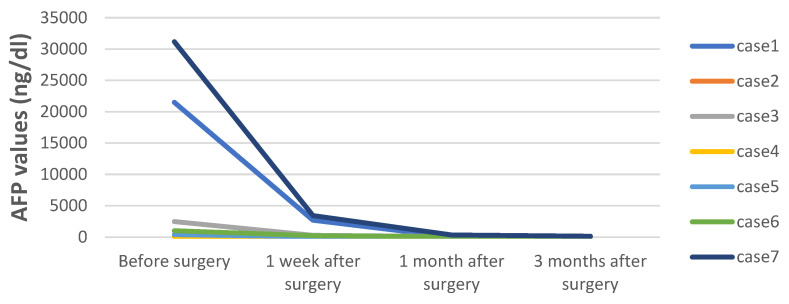
The trend of AFP levels in children with HB undergoing ICG fluorescence-guided laparoscopic surgery. It can be noted from the chart that AFP levels in all of the children involved decreased significantly after surgery, before finally returning to a normal level.

The average operation time was 359 min (range, 250–570 min) with an average intraoperative blood loss of 280 mL (range, 50–600 mL). The tumors were resected en bloc without rupture, air embolism, and hemorrhage, neither were there postoperative complications such as infection, hemorrhage, or liver failure. Two cases developed biliary fistulas after surgery, but were cured via non-surgical treatment with drainage. None of the cases had tumor implantation nor metastases in the Trocar ports. Abdominal CT showed no tumor recurrence one month post-operation, whereas the AFP level gradually returned to normal within six months after surgery (Figure 6). Postoperative chemotherapy was completed. No tumor residue, recurrence, or metastases were found by the hematologic test and whole-body PET/CT scan. The cases were followed up for 9–37 months (average, 24 months). There has been no recurrence or death so far.

## 4. Discussion

Radical liver resection is the most critical and effective treatment for patients with HB. With the various benefits that laparoscopy brings compared with open surgery such as being less traumatic, quick recovery, beautiful incision, etc., laparoscopy has become one of the most preferred surgical procedure in adult hepatectomy. Laparoscopic liver resection (LLR) accounts for more than 80% of hepatectomy in some internationally renowned medical centers [14,15]. However, LLR is rarely performed in children due to the lack of tactile sensation and the small volume of abdominal cavity. At present, open liver resection (OLR) is the preferred option in treating pediatric HB for a vast majority of medical centers. Nevertheless, more and more pediatric surgeons are showing interest in practicing laparoscopic HB removal [16,17,18] due to the rapid development of laparoscopic technology and equipment. Current studies show no significant difference in the 3-year tumor-free survival rate between open surgery and laparoscopy for HB [19,20]. Our previous studies have also shown that laparoscopic techniques are safe and feasible in the treatment of giant HB [21]. In addition, navigating laparoscopic HB resection with ICG fluorescence is advantageous due to the real-time display of tumor boundaries and small metastases. It can effectively avoid positive margins or tumor residues. Nonetheless, only one case of laparoscopic HB resection under ICG fluorescence navigation has been reported thus far, to the best of our knowledge. Henceforth, the main objective of this study was to summarize our experience in laparoscopic HB resection using fluorescence navigation.

ICG is a water-soluble, inert compound that can be used to evaluate liver function and navigate hepatectomy [22]. At present, ICG fluorescence is widely used in intraoperative tumor identification, surgical margin definition, and surgical navigation of hepatocellular carcinoma. Due to ICG being safe with good imaging effects, ICG fluorescence technology has been gradually applied in the treatment of HB among children [8,9,10,11,12,13]. For all cases involved in this study, no allergic reactions or serious side effects were noticed to be caused by ICG. Moreover, fluorescence laparoscopy not only has all the advantages of laparoscopy, but also possess the critical function of real-time fluorescence imaging navigation, which can provide accurate intraoperative information on tumor localization and boundary delineation. Moreover, the major downside of laparoscopic surgery, which is the lack of tactile sensation, could also be eliminated. These strengths of ICG fluorescence-guided laparoscopy drove us to perform tumor R0 resection with it. Postoperative pathological examination confirmed a negative tumor margin. No tumor residue, recurrence, or metastases were found among all patients involved in this study during follow-up visits for 9–37 months post-operation.

The timing and dosage of ICG injection before operation has a great influence on the effect of intraoperative visualization. However, there is currently no consensus among physicians on the optimal ICG timing and dosage for the fluorescence imaging of liver tumors in children. Administered doses range from 5 mg/body to 20 mg/body or from 0.25 mg/kg to 0.5 mg/kg [23]. A study showed that if ICG was injected intravenously, normal liver tissue would develop uniform fluorescence within 5–10 min and continue until 20–24 h after injection [24]. Yohei Yamada et al. recommended an interval between the injection of ICG (0.5 mg/kg) and the operation was approximately 72 h [25]. Given that children tend to have very little cirrhosis or other underlying liver disease, and that tumors tend to be highly differentiated, the seven cases in this study were administered with ICG 48 h before surgery at a dosage of 0.5 mg/kg, with all patients successfully achieving clear fluorescence visualization in their primary HB lesions. It is recommended for ICG to be injected 72–96 h prior to surgery to minimize the background fluorescence in the intestinal circulation, as this nonspecific fluorescence will dissipate by then [26]. However, we suggest that the timing for ICG administration should be evaluated based on the actual situation of the individual child. For younger children, the timing of administration can be appropriately advanced to 48 h before surgery due to rapid liver metabolism; as for children with poor liver function or even cirrhosis, the timing of administration can instead be appropriately delayed to approximately 96 h before surgery.

Tumor pathological type, degree of differentiation, and preoperative chemotherapy may also impact the effect of fluorescence imaging. Well-differentiated hepatocellular carcinoma tissue showed strong fluorescence on the cut surface since these tissues absorb ICG well, whereas poorly differentiated HCC and metastatic HCC resulted in poor imaging with low fluorescence intensity since they did not absorb ICG [23]. Fetal HB is the main subtype of HB, so it often possesses better differentiation characteristics, which consequently leads to full or partial fluorescence, similar to the characteristics of well-differentiated hepatocellular carcinoma. Nevertheless, fluorescence imaging of the tumor lesion in case no. 2 was not obvious and irregular, and also showed an unclear boundary of the lesion (Figure 2). Tumor necrosis due to the three cycles of hepatic artery embolization before operation was deduced as the possible reason, as this led to the lack of ICG uptake among liver cancer cells and liver cells in the necrotic area, causing the failure of some tumor areas to fluoresce.

Despite all the benefits that it brings, ICG fluorescence-guided surgery has also its own limitations, with one of it being false-positive results. Gotoh et al. found that strong fluorescence could be displayed by cirrhotic nodules and hepatic dysplasia nodules, with a false-positive rate of 40–50% and an accuracy rate of 65% [27]. A false-positive result in the case of ICG fluorescence-guided laparoscopy must be avoided to prevent unnecessary resections in patients. However, no detailed study has been carried out on false-positive results in the primary lesions of HB. No false-positive case was found among our patients. Another technical limitation of ICG fluorescence-guided surgery is that the fluorescence emitted by ICG can only penetrate 5–10 mm thick tissues [28]. As a result, deeper lesions cannot be properly visualized. Photoacoustic imaging technology, which can observe ICG accumulation using images obtained by intraoperative ultrasound examination simultaneously, is being developed to solve this problem [29]. Case no. 6 was 3 months old during surgery. Through this case, it was found that fluorescence laparoscopy worked even for a distance between the tumor and the liver surface that was slightly deeper than 10 mm, if combined with the imaging results from preoperative CT. Fluorescence imaging of the tumor was found by making a very shallow incision across the liver tissue, which was right above the tumor. Fluorescence-guided laparoscopic hepatectomy of Segment 8 can then be carried out successfully (Figure 4). At present, target cases need to be carefully selected. We also need to be prepared for the lack of fluorescence visualization in cases with deep tumors. Intraoperative B-ultrasound must be used to clarify the location of the tumor should fluorescence imaging fail to yield positive results during the procedure. On the other hand, if there is no intraoperative fluorescence imaging, the risk of cutting through the tumor may be relatively higher. The third limitation of ICG fluorescence imaging is that ICG may only display tumor tissues that are larger than 3 mm in diameter. Currently, no detailed reports are available on the effectiveness of fluorescence imaging in HB less than 3 mm in diameter.

It is worth mentioning that case no. 7 was only 13 days old during the surgery, making this patient possibly the youngest HB case to have underwent laparoscopic resection. The initial diagnosis of hepatoblastoma was not well-supported by the relatively low level of AFP in this patient. However, considering the location of the tumor in the left outer lobe of the liver and the small size of the tumor, preoperative chemotherapy was not recommended, but we proceeded directly to surgery instead. Additionally, this child also had combined right hydronephrosis, and a laparoscopic right dismembered pyeloplasty was performed three months after the HB resection surgery.

In general, the application of ICG fluorescence-guided laparoscopy in HB resection is safe and effective. It clearly displays tumor lesions, allowing for accurate lesion removal with real-time intraoperative navigation. Nevertheless, the limitations of this technology must also be taken note of. Its possible effect in improving postoperative outcomes remains to be clarified. Although the early recurrence rate was observed to decrease after the introduction of ICG fluorescence imaging in a study on HCC, no survival benefit has been observed [30]. Due to the lower incidence rate of HB compared to HCC, a larger number of cases needs to be collected and retrospectively analyzed before a conclusion can be drawn. The goal of possible future research is therefore set to determine whether fluorescence laparoscopy can actually improve the prognosis of HB.

## 5. Conclusions

ICG-guided fluorescence laparoscopic HB resection is safe and effective, yielding clear visualization of the tumor location, precise delineation of the tumor margin, and allows for the accurate removal of lesions with the aid of real-time navigation during operation.

## Figures and Tables

**Table 1:** Clinical, pathological, and surgical data of seven children who underwent ICG fluorescence-guided laparoscopic HB resection.

Case	Sex	Age at Surgery	PRETEXT	POST-TEXT	Chemotherapy	AFP at Surgery (ng/dL)	Tumor Diameter (mm)	Interval Time of ICG Injection/Surgery	ICG Dosage (mg/Kg)	Tumor Depth from Liver Surface	Surgical Procedure	Operation Time (min)	Pathology	Follow-up Days	Outcome
1	Male	14 M	III	II	4 C5VD	21,492	75	48 h	0.5	0mm	PH (S5,S6)	250	Fetal and embryonal	1127	Alive without disease
2	Male	22 M	III	II	3 C5VD	60.37	70	48 h	0.5	0mm	PH (S5,6,7)	295	Embryonal	1085	Alive without disease
3	Male	9 M	III	II	4 C5VD	2468	65	48 h	0.5	0mm	RH	410	Epithelial and mesenchymal	1012	Alive without disease
4	Male	36 M	III	II	3 CCD + 2 ICE	82.5	87	48 h	0.5	0mm	CBS	570	Fetal	779	Alive without disease
5	Male	13 M	III	II	4 C5VD	398.6	47	48 h	0.5	0mm	PH (S4a)	310	Fetal	448	Alive without disease
6	Male	3 M	II	I	2 C5VD	1025	25	48 h	0.5	11.5mm	PH (S8)	430	Epithelial and mesenchymal	259	Alive without disease
7	Female	13 D	I	I	None	31,178	30	48 h	0.5	0mm	LLS	250	Fetal	247	Alive without disease

RH, right hemihepatectomy; PH, partial hepatectomy; CBS, central bisegmentectomy; LLS, left lateral segmentectomy; M, month; D, days; Kg, kilogram; MM, millimeter; PRETEXT, pre-treatment extent of disease; POSTTEXT, post-treatment extent of disease; C5VD, cisplatin, 5-fluorouracil, vincristine and doxorubicin, ICE, ifosphamide, carboplatin, etoposide.

## Data Availability

The numerical datasets analyzed in this study are available from the corresponding author on reasonable request. The Digital Imaging and Communications in Medicine (DICOM) files cannot be made freely available due to privacy restrictions.

## References

[1] Meyers R., Hiyama E., Czauderna P., Tiao G.M. (2021). Liver tumors in pediatric patients. Surg. Oncol. Clin. N. Am..

[2] Busweiler L.A., Wijnen M.H., Wilde J.C., Sieders E., Terwisscha V.S.S., van Heurn L.W., Ziros J., Bakx R., Heij H.A. (2017). Surgical treatment of childhood hepatoblastoma in the netherlands (1990–2013). Pediatr. Surg. Int..

[3] Shi Y., Commander S.J., Masand P.M., Heczey A., Goss J.A., Vasudevan S.A. (2017). Vascular invasion is a prognostic indicator in hepatoblastoma. J. Pediatr. Surg..

[4] Narasaki H., Noji T., Wada H., Ebihara Y., Tsuchikawa T., Okamura K., Tanaka E., Shichinohe T., Hirano S. (2017). Intraoperative real-time assessment of liver function with near-infrared fluorescence imaging. Eur. Surg. Res..

[5] Ishizawa T., Fukushima N., Shibahara J., Masuda K., Tamura S., Aoki T., Hasegawa K., Beck Y., Fukayama M., Kokudo N. (2009). Real-time identification of liver cancers by using indocyanine green fluorescent imaging. Cancer-Am. Cancer Soc..

[6] Liberale G., Bourgeois P., Larsimont D., Moreau M., Donckier V., Ishizawa T. (2017). Indocyanine green fluorescence-guided surgery after iv injection in metastatic colorectal cancer: A systematic review. Eur. J. Surg. Oncol..

[7] Yamamichi T., Oue T., Yonekura T., Owari M., Nakahata K., Umeda S., Nara K., Ueno T., Uehara S., Usui N. (2015). Clinical application of indocyanine green (icg) fluorescent imaging of hepatoblastoma. J. Pediatr. Surg..

[8] Lake C.M., Bondoc A.J., Dasgupta R., Jenkins T.M., Towbin A.J., Smith E.A., Alonso M.H., Geller J.I., Tiao G.M. (2021). Indocyanine green is a sensitive adjunct in the identification and surgical management of local and metastatic hepatoblastoma. Cancer Med..

[9] Yamada Y., Hoshino K., Mori T., Kawaida M., Abe K., Takahashi N., Fujimura T., Kameyama K., Kuroda T. (2018). Metastasectomy of hepatoblastoma utilizing a novel overlay fluorescence imaging system. J. Laparoendosc. Adv. Surg. Tech..

[10] Souzaki R., Kawakubo N., Matsuura T., Yoshimaru K., Koga Y., Takemoto J., Shibui Y., Kohashi K., Hayashida M., Oda Y. (2019). Navigation surgery using indocyanine green fluorescent imaging for hepatoblastoma patients. Pediatr. Surg. Int..

[11] Cho Y.J., Namgoong J., Kwon H.H., Kwon Y.J., Kim D.Y., Kim S.C. (2021). The advantages of indocyanine green fluorescence imaging in detecting and treating pediatric hepatoblastoma: A preliminary experience. Front. Pediatr..

[12] Misa Y., Mio T., Norihiko K., Kumiko N., Masato S., Hiroaki G., Yukichi T. (2022). Clinicopathological study of surgery for pulmonary metastases of hepatoblastoma with indocyanine green fluorescent imaging. Pediatr. Blood Cancer..

[13] Yuanchao S., Manna Z., Jiahao L., Tianbao T., Jiliang Y., Jing P., Chao H., Yan Z., Tianyou Y. (2022). Clinical Application of Indocyanine Green Fluorescence Imaging in the Resection of Hepatoblastoma: A Single Institution’s Experiences. Front. Surg..

[14] Koffron A.J., Auffenberg G., Kung R., Abecassis M. (2007). Evaluation of 300 minimally invasive liver resections at a single institution: Less is more. Ann. Surg..

[15] Reddy S.K., Tsung A., Geller D.A. (2011). Laparoscopic liver resection. World J. Surg..

[16] Kwon H., Lee J.Y., Cho Y.J., Kim D.Y., Kim S.C., Namgoong J. (2019). How to safely perform laparoscopic liver resection for children: A case series of 19 patients. J. Pediatr. Surg..

[17] Urade T., Sawa H., Iwatani Y., Abe T., Fujinaka R., Murata K., Mii Y., Man-i M., Oka S., Kuroda D. (2020). Laparoscopic anatomical liver resection using indocyanine green fluorescence imaging. Asian J. Surg..

[18] Yada K., Ishibashi H., Mori H., Shimada M. (2014). Laparoscopic resection of hepatoblastoma: Report of a case. Asian J. Endosc. Surg..

[19] Yuan X.J., Wang H.M., Jiang H., Tang M.J., Li Z.L., Zou X., Fang Y.J., Pan C., Tou J.F., Zhang K.R. (2016). Multidisciplinary effort in treating children with hepatoblastoma in china. Cancer Lett..

[20] Hiyama E., Hishiki T., Watanabe K., Ida K., Yano M., Oue T., Iehara T., Hoshino K., Koh K., Tanaka Y. (2016). Resectability and tumor response after preoperative chemotherapy in hepatoblastoma treated by the Japanese study group for pediatric liver tumor (jplt)-2 protocol. J. Pediatr. Surg..

[21] Wu Y., Zeng L., Qiu R., Zhang J., Su J., Liao M., Deng X. (2021). Two-stage laparoscopic resection of giant hepatoblastoma in infants combined with liver partial partition and artery ligation. World J. Surg. Oncol..

[22] Landsman M.L., Kwant G., Mook G.A., Zijlstra W.G. (1976). Light-absorbing properties, stability, and spectral stabilization of indocyanine green. J. Appl. Physiol..

[23] Nakaseko Y., Ishizawa T., Saiura A. (2018). Fluorescence-guided surgery for liver tumors. J. Surg. Oncol..

[24] Kitagawa N., Shinkai M., Mochizuki K., Usui H., Miyagi H., Nakamura K., Tanaka M., Tanaka Y., Kusano M., Ohtsubo S. (2015). Navigation using indocyanine green fluorescence imaging for hepatoblastoma pulmonary metastases surgery. Pediatr. Surg. Int..

[25] Yamada Y., Ohno M., Fujino A., Kanamori Y., Irie R., Yoshioka T., Miyazaki O., Uchida H., Fukuda A., Sakamoto S. (2019). Fluorescence-Guided Surgery for Hepatoblastoma with Indocyanine Green. Cancers.

[26] de Graaf W., Bennink R.J., Vetelainen R., van Gulik T.M. (2010). Nuclear imaging techniques for the assessment of hepatic function in liver surgery and transplantation. J. Nucl. Med..

[27] Gotoh K., Yamada T., Ishikawa O., Takahashi H., Eguchi H., Yano M., Ohigashi H., Tomita Y., Miyamoto Y., Imaoka S. (2009). A novel image-guided surgery of hepatocellular carcinoma by indocyanine green fluorescence imaging navigation. J. Surg. Oncol..

[28] Lim C., Vibert E., Azoulay D., Salloum C., Ishizawa T., Yoshioka R., Mise Y., Sakamoto Y., Aoki T., Sugawara Y. (2014). Indocyanine green fluorescence imaging in the surgical management of liver cancers: Current facts and future implications. J. Visc. Surg..

[29] Hsu C.W. (2019). Lymph node mapping and anastomosis evaluation by visera elite ii^®^, a novel surgical endoscope system, with infrared fluorescence imaging during laparoscopic rectal cancer surgery—A video vignette. Color. Dis..

[30] Morita Y., Sakaguchi T., Unno N., Shibasaki Y., Suzuki A., Fukumoto K., Inaba K., Baba S., Takehara Y., Suzuki S. (2013). Detection of hepatocellular carcinomas with near-infrared fluorescence imaging using indocyanine green: Its usefulness and limitation. Int. J. Clin. Oncol..

